# Levels of Heterochiasmy During *Arabidopsis* Development as Reported by Fluorescent Tagged Lines

**DOI:** 10.1534/g3.120.401296

**Published:** 2020-04-22

**Authors:** Ramswaroop Saini, Amit Kumar Singh, Geoffrey J. Hyde, Ramamurthy Baskar

**Affiliations:** * Department of Biotechnology, Indian Institute of Technology–Madras, Bhupat and Jyoti Mehta School of Biosciences, Chennai 600 036, India,; † Department of Biotechnology, Kalinga University, Raipur, Chhattishgarh 492101 India,; ‡ School of Plant Sciences and Food Security, Tel Aviv University, Tel Aviv 6997801, Israel, and; § Independent Researcher, Randwick, New South Wales, Australia

**Keywords:** *Arabidopsis thaliana*, meiotic recombination, parental age, centromere, crossing over, heterochiasmy

## Abstract

Crossing over, the exchange of DNA between the chromosomes during meiosis, contributes significantly to genetic variation. The rate of crossovers (CO) varies depending upon the taxon, population, age, external conditions, and also, sometimes, between the sexes, a phenomenon called heterochiasmy. In the model plant *Arabidopsis thaliana*, the male rate of all crossover events (mCO) is typically nearly double the female rate (fCO). A previous, PCR-based genotyping study has reported that the disparity decreases with increasing parental age, because fCO rises while mCO remains stable. We revisited this topic using a fluorescent tagged lines approach to examine how heterochiasmy responded to parental age in eight genomic intervals distributed across the organism’s five chromosomes. We determined recombination frequency for, on average, more than 2000 seeds, for each interval, for each of four age groups, to estimate sex-specific CO rates. mCO did not change with age, as reported previously, but, here, fCO did not rise, and thus the levels of heterochiasmy were unchanged. We can see no methodological reason to doubt that our results reflect the underlying biology of the accessions we studied. The lack of response to age could perhaps be due to previously reported variation in CO rate among accessions of *Arabidopsis*.

During meiotic crossing over, homologous chromosomes align and exchange paternally and maternally derived DNA. Crossovers (CO) are one of the main sources of variation in sexually reproducing organisms, and as such, the rate at which they occur has considerable evolutionary significance (Ritz *et al.* 2017; Stapley *et al.* 2017). If the rate is too low, the organism has less chance of adaptation; if too high, an already effective genotype runs the risk of disruption. While the rate of crossovers can vary across taxa, populations, and between and within individuals, the possible scale of variation across these various levels appears remarkably constrained (Ritz *et al.* 2017). Nevertheless, the scope for some degree of CO rate variation exists for individual organisms, and is of practical importance, both medically and economically. For example, the frequencies of several forms of human chromosomal number abnormalities correlate with changed rates of CO, relative to those typical of younger women (Hussin *et al.* 2011; Alves *et al.* 2017). In plant breeding, the development of ‘elite’ genotypes depends on meiotic COs that allow the accumulation of desirable traits, and much research is focused on finding ways to increase local CO rates (Wijnker and Dejong 2008; Crismani *et al.* 2013; Fernandes *et al.* 2018).

Interestingly, it is often not just the overall rate of CO that is important, but also the ratio of the male and female rates of CO (henceforth, mCO and fCO). In many taxa, these two rates differ to a greater or lesser extent, a phenomenon called heterochiasmy (Ritz *et al.* 2017; Stapley *et al.* 2017). In true heterochiasmy, the ratio between the rates of the more and less recombinative sexes can vary from 1.035 to 14 (Ritz *et al.* 2017). Evidence indicates that, for a heterochiasmatic species, the sex that has the lower rate of CO will be the one for which genetic stability in the haploid phase is most likely to be critical to the future organism’s fitness (Lenormand 2003; Lenormand and Dutheil 2005; Stapley *et al.* 2017). In *Arabidopsis*, for example, its high self-pollination rate (95%; (Charlesworth and Vekemans 2005) suggests that the female haploid phase is most critical, thus possibly explaining why fCO has the lesser value (Lenormand 2003; Lenormand and Dutheil 2005). The ratio of mCO:fCO in young *Arabidopsis* seedlings is typically about 1.8 (Toyota *et al.* 2011; Giraut *et al.* 2011).

As well as having evolutionary drivers, both the overall, and sex-specific, CO rates, and also mCO:fCO, are influenced by age and extrinsic stressors such as temperature, pathogens, chemical exposure, and lack of nutrients (Hayman and Parsons 1962; Francis *et al.* 2007; Toyota *et al.* 2011; Hussin *et al.* 2011; Martin *et al.* 2015; Halldorsson *et al.* 2016; Li *et al.* 2017; Modliszewski and Copenhaver 2017; Saini *et al.* 2017; Stapley *et al.* 2017). The effect of age on mCO:fCO, and its mechanistic basis, has been much studied in humans because the changed rates of CO implicated in the chromosomal number abnormalities mentioned above mostly occur in older women (Hussin *et al.* 2011; Chiang *et al.* 2012; Nagaoka *et al.* 2012; Alves *et al.* 2017).

For plant CO, much less is known about age x sex interactions. For example, with respect to the influence of age on patterns of heterochiasmy in *Arabidopsis*, there has only been one study (Toyota *et al.* 2011). They used PCR-based genotyping, examining 343 markers across the five chromosomes, and found that the extent of heterochiasmy in primary shoots decreased across the two time-points chosen for the study. Interestingly, this was solely due to an increase in fCO; there was no change in mCO. Other studies, using fluorescent markers that allow the detection of recombination in pollen, have examined the response of mCO to either age (Li *et al.* 2017), developmental position (Francis *et al.* 2007) or both (Li *et al.* 2017). Li *et al.* (2017) found that while mCO in primary shoots did not significantly change with age for markers in five of nine genomic intervals (thus in agreement with the earlier results of Toyota *et al.* 2011), the rates did significantly increase in two intervals.

In this study, we revisit the response of heterochiasmy to age, using a seed-based, fluorescent tagged lines approach (Melamed-Bessudo *et al.* 2005; Pecinka *et al.* 2011; Wu *et al.* 2015; van Tol *et al.* 2018) to determine the frequency of recombinant seeds, as an estimator of CO rate in eight intervals that cover all five chromosomes of *Arabidopsis*. Plants were sampled at four time points that cover the full reproductive duration of the *Arabidopsis* main shoot. We found that mCO did not change with age, as reported in the earlier study (Toyota *et al.* 2011), but in contrast to their finding, we saw no rise in fCO. Thus, the level of heterochiasmy was unaffected by age. We can see no methodological reason for doubting the validity of our finding, and suggest that biological differences between the accessions used in the two studies might have resulted in the different outcomes.

## Materials and methods

### Plant growth conditions

Freshly harvested *Arabidopsis* seeds from Columbia or detector lines (described below) were surface sterilized with 70% ethanol, followed by 0.5% bleach treatment for 3 min. Subsequently, the seeds were washed thrice with sterile water and plated on autoclaved Murashige and Skoog media (MS, with 3% sucrose), pH 5.7, containing 0.05% Plant Preservative Mixture (Biogenuix Medsystem Pvt. Ltd., New Delhi, India) and incubated at 4° in dark conditions, for synchronized germination. After 48 h, the plates were shifted to a seed germination chamber, with a uniform light intensity of 8000 lux units (16-h light/8-h dark cycle). The temperature of the chamber (Percival CU-36L6) was maintained at 22° with a constant humidity of 80%. Three-week old seedlings were transferred from MS plates to soil and grown inside a plant growth chamber (Percival AR-36L3). The soil had equal proportions of garden soil, peat, perlite, and vermiculite (Keltech Energies Ltd., Bangalore, India).

### Arabidopsis detector lines used to estimate CO rates

To estimate CO rates, eight different detector lines covering at least one marker in each of the five chromosomes were used to measure the number of recombinant seeds. The detector lines Col3-4/20, 3158 and 3162 were kind gifts from Avraham A. Levy (Department of Plant Sciences, Weizmann Institute of Science, Israel), (Melamed-Bessudo *et al.* 2005). Another set of detectors, the traffic lines CTL1.2, CTL1.18, CTL2.4, CTL4.7 and CTL5.17 was obtained from the Arabidopsis Biological Resource Center (Ohio State University, USA), (Wu *et al.* 2015) (Table 1). In all the lines, the eGFP and dsRed markers are driven by a seed-specific napin promoter. The detector lines, homozygous for both markers were crossed with Columbia plants, and the seeds obtained (heterozygous for both eGFP and dsRed) were used in the subsequent experiments.

**Table 1:** Estimated sex-specific CO rates, and estimated and predicted ratios

Line Name	Chr. No.	Estimated average CO rates in males (mCO)	Estimated average CO rates in females (fCO)	Estimated mCO:fCO (All ages combined)	Predicted mCO:fCO
CTL1.2	1	33.44	28.24	1.184	1.20
CTL1.18	1	17.95	7.81	2.30	3.25
CTL2.4	2	17.84	13.41	1.33	2.32
Col3-4/20	3	24.04	8.90	2.67	3.49
3158	3	25.66	10.88	2.33	3.06
CTL4.7	4	23.17	8.67	2.67	3.21
3162	5	19.33	14.73	1.22	1.28
CTL5.17	5	16.62	5.21	3.20	2.46

### Investigating parental age effect on CO rates

To examine the influence of parental age on CO rates, plants of the detector lines and Columbia plants, of four different ages (40, 45, 50 and 55 DAS (days after sowing), were emasculated 48 h before pollination and reciprocally crossed with each other. Different colored threads were used to mark emasculated and pollinated flowers of different age groups. For each age, approximately 20 to 30 crosses were performed in replicate (3, 6 and 9). To score recombination during megaspore formation, we used emasculated flowers from the detector lines and crossed them with pollen from Columbia plants. Similarly, to estimate recombination rates during microspore formation, we used a detector line as the pollen donor for emasculated flowers of Columbia.

### Calculation of recombination frequency as an estimator of CO rates

The segregation of eGFP and dsRed markers (an indication of recombination during micro- or mega-sporogenesis in the detector line parent), was analyzed by the manual counting of seeds. Seeds were placed on a glass slide and analyzed under a Nikon Stereozoom Microscope (SMZ 1000) equipped with filters specific for both eGFP and dsRed (SZX-MG for GFP and SZX-MGFPA for RFP). Images were captured for eGFP and dsRed separately and then both the images merged to identify the recombinant and non-recombinant seeds. An average of over 2200 seeds per line, age, and sex were examined (Supplementary Tables S1-S9). Frequencies were estimated based on the segregation of eGFP and dsRed markers. Of the four types of seeds obtained, seeds that fluoresce only red or only green were counted as having undergone recombination, while the seeds that fluoresce for both red and green as well as those that do not fluoresce at all, were counted as not having undergone recombination (Supplementary Tables S10-S17). Frequencies were calculated based on the formula:

$$\text{Recombination frequency}=\frac{\left(\text{R}+\text{G}\right)}{\left(\text{R}+\text{G}+\text{RG}+\text{NFS}\right)}\times 100$$

R: dsRed-only expressing seeds; G: eGFP only expressing seeds; RG: Seeds expressing both dsRed and eGFP; NFS- non-fluorescent seeds. The formula is a variation of one used previously (Melamed-Bessudo *et al.* 2005), adjusted to accommodate our use of homozygous x homozygous parents. The recombination frequency, which is a dimensionless number, is used here as an adequate estimator of the relative values of sex-specific CO rates, *i.e.*, mCO and fCO, and thus of the ratio of the two. In the *The issue of non-detection* sub-section (*Discussion)*, we consider the adequacy of this approach at length. Note that the absolute value of each rate as computed here (*i.e.*, being non-dimensional) cannot be compared with the rates derived in a PCR-based genotyping study.

### Statistical analysis

Recombination frequencies follow a normal distribution and hence, a Gaussian generalized linear model (GLM) with identity link function was used (Nelder and Wedderburn 1972). The linear predictors were either the different ages, or the sex, of the detector-line parent. In all GLMs, the data from groups were compared. Correction for multiple testing was done to maintain the family-wise error rate at 5% (Gabriel 1969). Therefore, the *P* values were adjusted with a single-step method that considered the joint multivariate *t* distribution of the individual test statistic (Bretz *et al.* 2016). The results were reported with the two-sided *P* values adjusted for multiple comparisons (Singh *et al.* 2015). All statistical analyses were carried out in R (*Pinheiro et al.* 2014). To adjust the *P* values for multiple testing, the R package multcomp was used with the test specification ‘single-step’(Bretz *et al.* 2016). Graphs were produced using GraphPad Prism 8.

### Data availability

Reagent and data available upon request. Supplemental material available at figshare: https://doi.org/10.25387/g3.12119022.

## Results

### Heterochiasmy in eight intervals of Arabidopsis was unaffected by parental age

**Figure 1:**
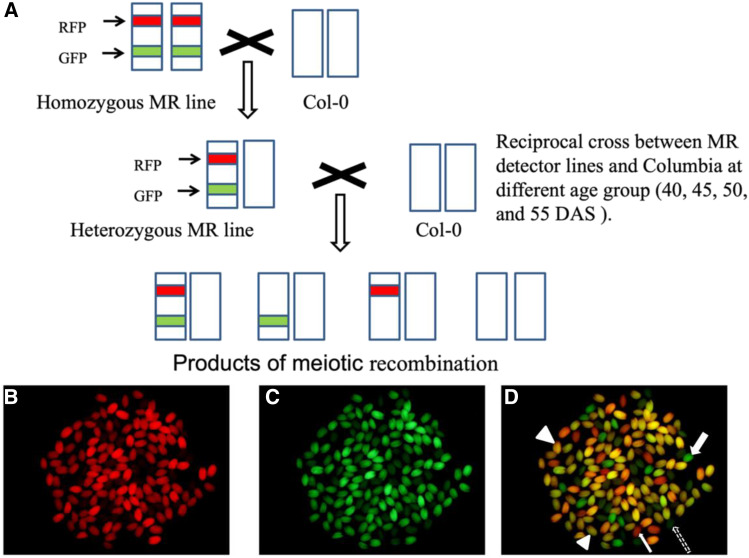
(A) A reciprocal cross between a heterozygous detector line and a Columbia plant results in seeds with one of four fluorescence patterns: a blend of red and green; only green; only red; no fluorescence. (B) A sample of seeds observed using a dsRed filter. Recombinant seeds exhibit variable fluorescence intensity in the red range, but non-recombinant seeds are dark (by comparison with same sample, shown in C and D). (C) The same sample of seeds observed using an eGFP filter. Recombinant seeds exhibit variable fluorescence intensity in the red range, but non-recombinant seeds are dark (by comparison with same sample, shown in B and D). (D) Merged image of B and C showing the four different patterns of fluorescence. Scoring began with the merged image, and we scored those seeds that clearly belonged to one of these four categories (1) red marker only; *e.g.*, thin continuous arrow; (2) green marker only; *e.g.*, thick continuous arrow; (3) both markers (gold/amber color); *e.g.*, large arrowhead; and (4) seeds without marker fluorescence; *e.g.*, thin dotted arrow. (Note that seeds in the latter category naturally have a very low level of green autofluorescence). A small number of seeds were uncertain, *e.g.*, the one marked with a small arrowhead. The categorization of these could be decided by comparing the same seed in the individual red and green filter images. For example, the last-mentioned seed is clearly fluorescent in both B and C, so it was scored as having both markers.

**Figure 2:**
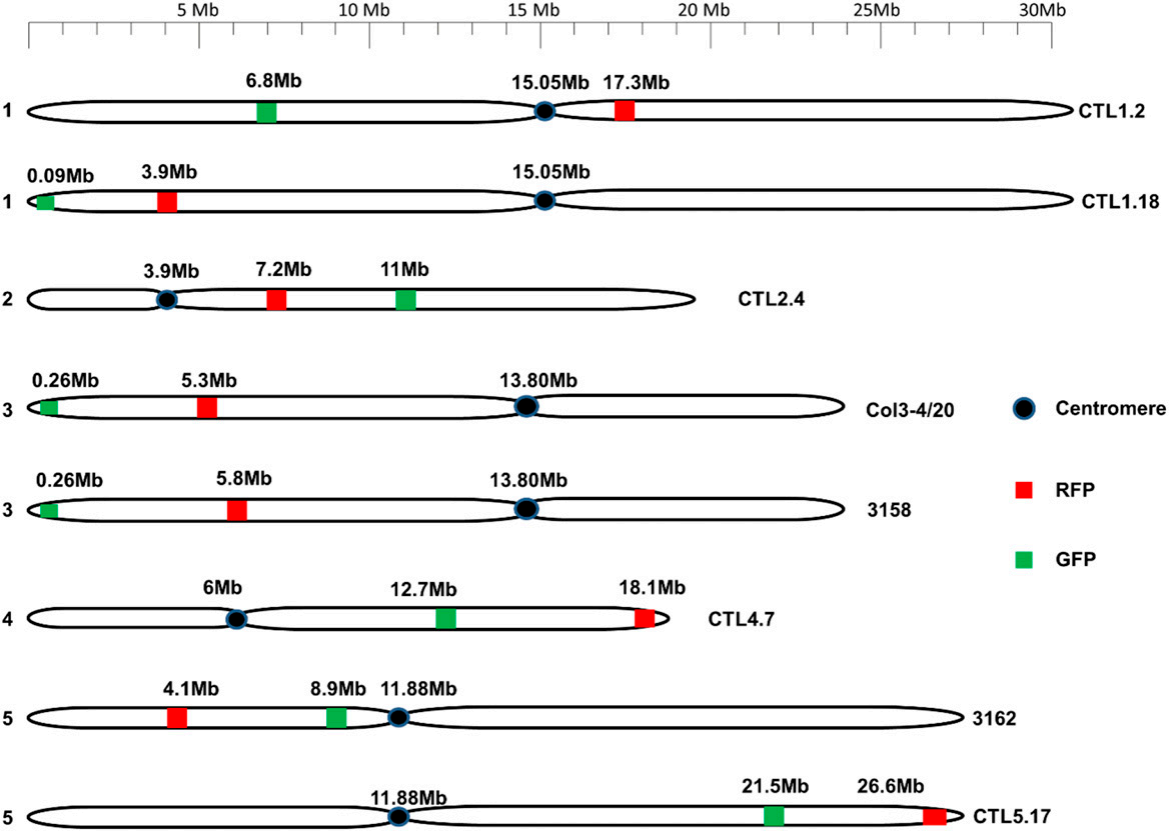
Physical maps of the chromosomes showing the location of the inter-marker intervals in the detector lines tested. Positions of eGFP and dsRed were drawn on the physical map using the chromosome map tool of The Arabidopsis Information Resource (TAIR).

Using a set of eight *Arabidopsis* detector lines, we examined the influence of parental age on male and female recombination frequencies. The eight detector lines heterozygous for both eGFP/dsRed were reciprocally crossed with Columbia wild type plants, with both parents being one of four ages (40, 45, 50, and 55 DAS) and recombination frequencies were examined in the collected seeds (Figure 1). The eight intervals were distributed across all five chromosomes, and varied in length and the degree of overlap with subtelomeric or pericentomeric regions (Figure 2; Supplementary Table S20). One interval (in line CTL1.2) spanned the centromere.

**Figure 3:**
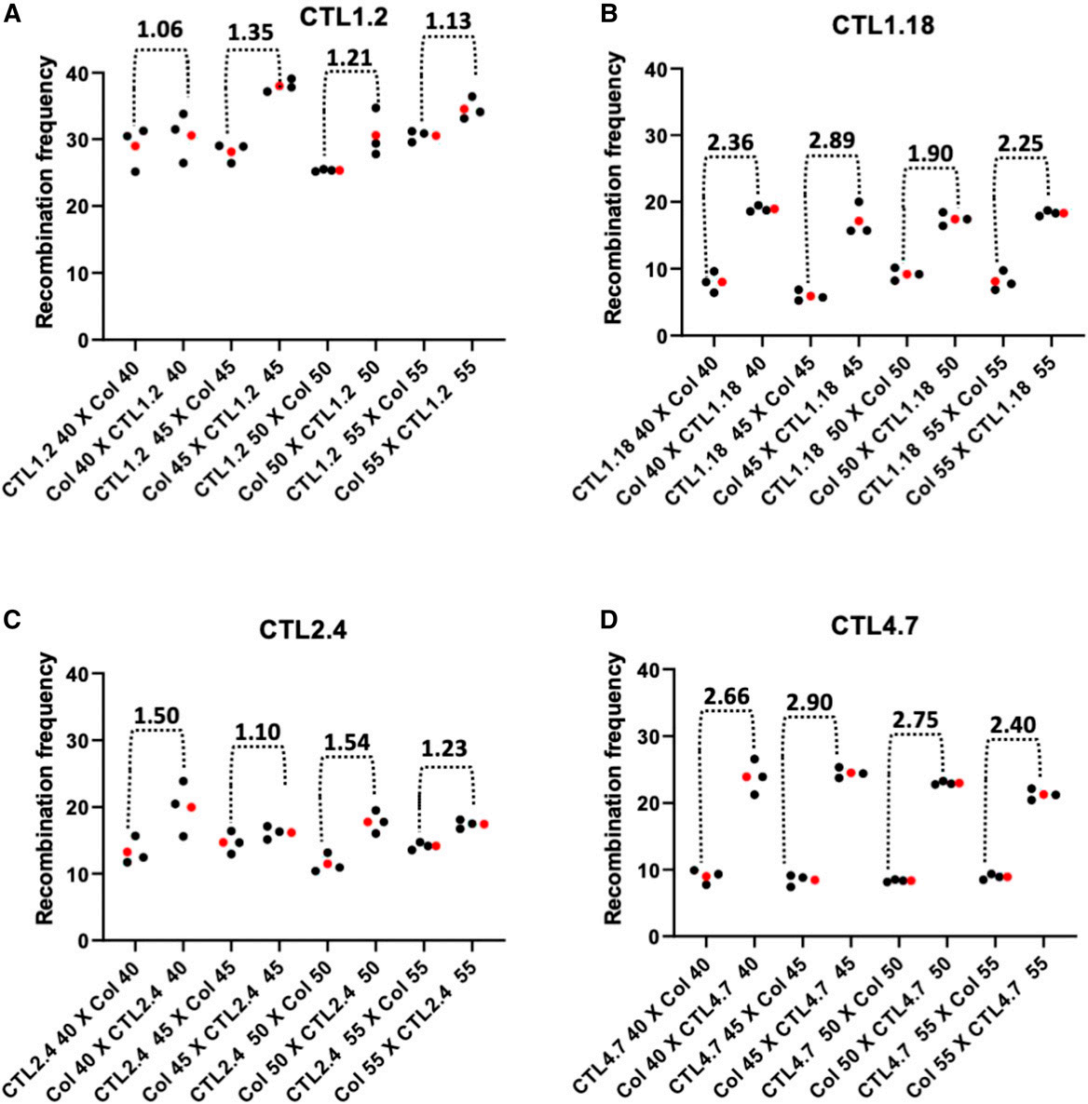

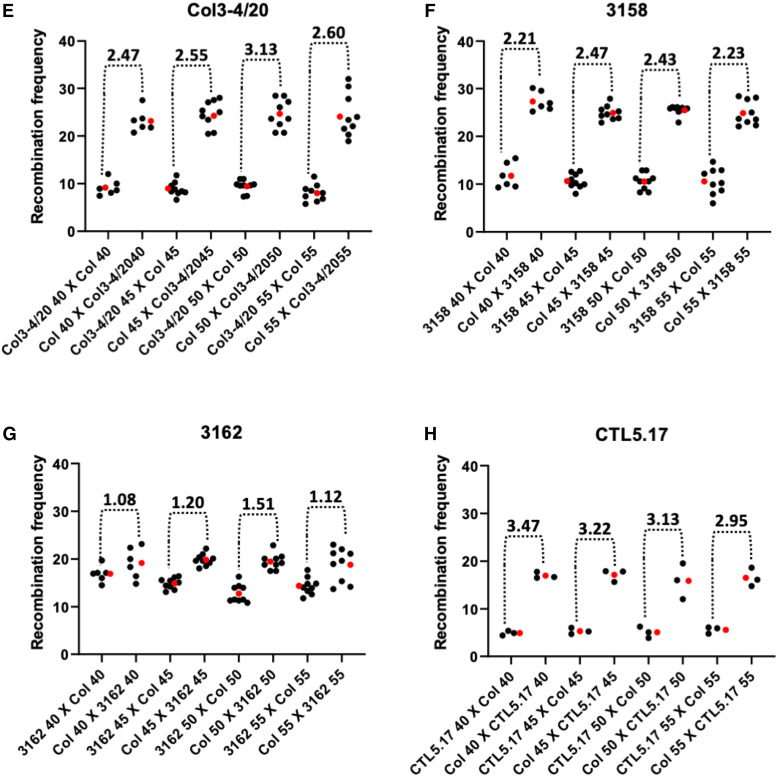
(A-H).Parental age did not affect recombination frequencies or sex ratios: Reciprocal crosses between detector lines CTL1.2, CTL1.18, CTL2.4, CTL4.7, Col3-4/20, 3158, 3162, CTL5.17 and Columbia plants (of 40, 45, 50 and 55 DAS). Each dotted bracket spans two clusters of data points (comprising the full set of data points for one age category); the first cluster is of female recombination frequencies, the second corresponds to those of male meiosis. The number above each bracket shows the average ratio of the male and female frequencies (*i.e.*, an estimator of mCO:fCO) for each age category. The graphs represent individual replicates (black dots) and mean value (red dots) of the recombination frequencies. GLM was used for detecting significant difference and *P* values were corrected for multiple testing (Supplementary Tables S10-S19).

For each of the eight intervals, there was no significant change in the estimated ratio, mCO:fCO, as the ages of the male and female parents were increased (Figure 3). Neither did the two individual rates that are used to calculate the ratio (*i.e.*, mCO; and fCO) vary with age (Figure 3; Supplementary Tables S10-S19).

The average heterochiasmic ratios, mCO:fCO, that we have estimated can also be compared with those predicted by analysis of data published previously (Table S2 in Giraut *et al.* (2011). Their genome-wide study reported the rates of mCO and fCO at 380 shared locations across all five *Arabidopsis* chromosomes, demonstrating remarkable variation in both rates from location to location. In Table 1 we present the estimated rates and ratios (all ages combined), and the predicted ratios, based on our interval-based analysis (Supplementary File S1) of the location-based data presented in their Table S2 (Giraut *et al.* 2011). The predicted values show only a weak congruence, in terms of their magnitudes relative to each other, with those that we measured.

The table shows the estimated sex-specific CO rates and ratios for each of the eight intervals studied here. The data for all age categories were combined because there was no significance difference across the four ages. The final column shows the ratios for the same intervals, based on our analysis of the data provided in Table S2 of Giraut *et al.* (2011). That data provides location-based male and female CO rates for 11 to 33 points per interval. Here, for each interval, the ratio was calculated using the average male or female CO rate across the interval, multiplied by the length of the interval (as per the values shown in our Supplementary File S1).

### Levels of heterochiasmy in eight intervals of Arabidopsis varied with the interval studied

The ratio mCO:fCO did, however, vary significantly on an interval by interval basis (Figure 3; Table 1). For example, the ratio of the average mCO:average fCO (across all four ages for any one interval) varied between 1.18 (CTL1.2) and 3.20 (CTL5.17; Table 1). These two lines also exhibited the most extreme values for both of the individual rates, mCO and fCO. CTL1.2, which had the lowest ratio, had the highest individual rates (mCO: 33.44; fCO: 28.24); while CTL5.17, which had the highest ratio, had the lowest rates (mCO: 16.62; fCO: 5.21) (Table 1).

The relatively higher values of the estimated male and female CO rates of detector line CTL1.2 were also predicted by our analysis of the data from Table S2 of Giraut *et al.* (2011). A high CO rate in an interval can be the consequence of the number and strength of its CO hotspots and/or the length of the interval. In the case of CTL1.2, for example, the estimated high male and female rates are due to the interval being two to three times longer than any of the others. This interval also had the highest predicted male and female rates of all the intervals (Supplementary File S1).

The pattern of high or low sex ratios can also be predicted from our calculations of the percentage overlap that intervals have with the subtelomeric and pericentromeric regions. For example, the only intervals with an mCO:fCO of less than 2.0 (*i.e.*, CTL1.2; CTL2.4; 3162; Table 1), are those that have no overlap with a subtelomeric region (Table 1; Figure 2), the latter being known for a concentration of male CO hotspots (Giraut *et al.* 2011).

## Discussion

Our results provide new insights into a previous finding that heterochiasmy in *Arabidopsis* decreases with age, in each of the organism’s five chromosomes (Toyota *et al.* 2011). In this study, only the second to address the topic, we used the fluorescent tagged lines (FTL) approach (instead of PCR-based genotyping) to score recombination frequencies, and found no change in the level of heterochiasmy in the eight intervals studied. As we argue below, we can see no reason why this aspect of our methodology reduces the reliability of our findings. The accessions we used, however, were also different from those used previously, and this is perhaps a more likely source of the different outcomes.

### The reliability of the two-marker FTL approach, as used in this study

The two-marker FTL approach has previously been used or recommended for studying meiotic recombination in *Arabidopsis* (Melamed-Bessudo *et al.* 2005; Pecinka *et al.* 2011; Wu *et al.* 2015; van Tol *et al.* 2018). The particular benefit of the FTL approach is that it facilitates mass screening of seeds. Here, we discuss some of the possible limitations of the approach, and argue that any given limitation is either not inherently serious, or its impact can be avoided.

### The issue of non-detection

The most widespread concern regarding the two-marker FTL approach, compared to the use of PCR-based genotyping, is this: A seed with two or more CO in the interval of interest will have the same fluorescence pattern as one in which only a single CO has occurred. Thus, the additional CO will not lead to an increased count of, for example, recombinant seeds, as would happen when the PCR-based approach is used (*e.g.*, noted by van Tol *et al.* 2018). This has prompted some studies of other systems to use three fluorescent markers (*e.g.*, (Francis *et al.* 2007), because this allows the scorer to distinguish instances of two CO in the interval of interest from those involving just one. Given that instances of more than two CO in an *Arabidopsis* chromosome are rare (in female meiosis of young plants, 0–1%; male: 1–9%; as per the data in Toyota *et al.* 2011), this has clearly been seen to provide a reasonable solution to the perceived problem.

But, is the use of two markers significantly less reliable than three, and more importantly, is the approach as a whole significantly less reliable than PCR-based genotyping? For studies like this, which are focused on calculating ratios of CO, we believe not, because the extent of underestimation of CO events (within the interval) is very low, as long as the interval length is relatively short.

The first point is that, in the typical interval-based FTL study, the vast majority of additional CO that occur in the interval of interest *will add* to the score of recombinant seeds. The non-detection of additional CO will only occur when an additional CO has occurred within the same interval as the initial CO event. How likely are these simultaneous events? If the interval is the entire chromosome, then the probability is 1, and this is indeed a problem. Interval lengths are, however, typically much shorter; for example in our study, for 7 of the 8 intervals used, the average length was 4.71Mb, about 20% of the average chromosomal length.

Within intervals of this length, CO interference will reduce the probability of non-detection sufficiently to allay concerns about the use of the two-marker approach. The average distance between double CO events in *Arabidopsis* has been reported as 11.58Mb (Yelina *et al.* 2015), and an analysis of the violin plot of Figure 4D of that study indicates that only about 10% of double CO occurred within 5Mb or less of each other. Regarding our single long interval (CTL1.2), analysis of the same violin plot indicates that about 40% of double CO would occur within an interval of its length, *i.e.*, 10.5Mb.The average distance between double CO events has also been estimated for chromosome 4, and reported as 1.58 times the expected (random) distance, the latter being one third of the length of the chromosome, when measured in centimorgans (Drouaud *et al.* 2006).

The second important point is that non-detection will affect both male and female rates, and, together with the fact that non-detection is rare, this means that the use of only two markers will have minimal effect on the measured ratios (mCO:fCO), compared to ratios estimated using three fluorescent markers, or PCR-based genotyping. The magnitude of the non-detection effect can be estimated by using the numbers provided in Table 5 of Toyota *et al.* (2011). When a non-detection level of 10% is used to recalculate the numbers of all CO additional to the initial CO, the change in mCO:fCO with age is reduced by only 1% (Supplementary File S2). Also, the possibility of this effect reducing the magnitude of the ratio should itself be reduced by the fact that CO interference levels are higher in female meiosis in *Arabidopsis* (Drouaud *et al.* 2007), and thus relatively fewer CO additional to the initial CO should go undetected in female meiosis (compared to the male).

In summary, the use of the two-marker FTL approach should itself not have prevented us from observing any reduction in mCO:fCO with age. Our argument that it is appropriate for a ratio-focused study like this should not, however, be seen as suggesting that the approach is equally useful for other purposes. We would not have even been in a position to evaluate its reliability if the PCR-based genotyping study (Toyota *et al.* 2011) had not established benchmark values for the proportion of double and triple CO within the overall recombination landscape.

### The issue of statistical reliability

As mentioned previously, the only discrepancy between our findings and those of Toyota *et al.* (2011) is that that we did not see a rise in fCO. Given that the rise in the number of female CO noted in the previous study was quite small (about 8%, for all chromosomes combined; see their Table 5), it is clear that studies of this phenomenon must be statistically robust. In our study, we exploited the opportunity for mass screening offered by the FTL approach: the average number of seeds counted for each interval, at each of the four time-points, was about 2200. Our study embraced the duration of the life-cycle covered by the two ages used in Toyota *et al.* (2011), but also included two intermediate time-points, and our experiments always featured replication.

It was also important to include intervals that span chromosomal regions where female CO are most frequent, since it is there that any mild trends are probably more likely to be detected. The chromosomes of *Arabidopsis* are known to have hot and cold spots, wherein female (or male) CO are respectively more or less likely; and there are also broader hot and cold zones, wherein hot or cold spots are, respectively more or less clustered (Drouaud *et al.* 2006; Giraut *et al.* 2011). Hot zones of male CO are subtelomeric, while female CO hot zones are nearer the centromere (Giraut *et al.* 2011). Our study included the full range of distributional scenarios (*i.e.*, subtelomeric, pericentromeric; neither subtelomeric nor pericentromeric), as was reflected in the marked variation in the measured values of mCO:fCO and in the values predicted by our analysis of the data of Giraut *et al.* (2011). Nevertheless, not only did we not see any significant rise in fCO with age in any interval, we saw no evidence even of any rising trend.

### Conclusions

We believe the methodology used in this study was fit for purpose. The fact that fCO, and thus mCO:fCO, remained at the same level throughout the life-cycle, in contrast to the patterns reported in the previous study, might therefore reflect differences in the underlying biology. Toyota *et al.* (2011) used two different types of ecotypes in their study (Columbia and Ler). They created the heterozygous detector line (for markers) by crossing between these two ecotypes, while in our study we generated heterozygous detector lines by crossing an homozygous detector line (in a Columbia background) with wild type Columbia plants (a pure line). Genetic background and heterozygosity have previously been reported as influences on recombination rates in *Arabidopsis* (Barth *et al.* 2001; López *et al.* 2012). Whether these differentially affect male and female rates, and their response to age, is worthy of further study.
